# Biosynthesis of Commodity Chemicals From Oil Palm Empty Fruit Bunch Lignin

**DOI:** 10.3389/fmicb.2021.663642

**Published:** 2021-04-09

**Authors:** Tat-Ming Lo, In Young Hwang, Han-Saem Cho, Raissa Eka Fedora, Si Hui Chng, Won Jae Choi, Matthew Wook Chang

**Affiliations:** ^1^NUS Synthetic Biology for Clinical and Technological Innovation (SynCTI), National University of Singapore, Singapore, Singapore; ^2^Synthetic Biology Translational Research Program, Yong Loo Lin School of Medicine, National University of Singapore, Singapore, Singapore; ^3^Department of Biochemistry, Yong Loo Lin School of Medicine, National University of Singapore, Singapore, Singapore; ^4^Institute of Chemical and Engineering Sciences, Agency for Science, Technology and Research, Singapore, Singapore

**Keywords:** direct biosynthesis, commodity chemicals, metabolic engineering, oil palm empty fruit bunch, lignin, synthetic biology

## Abstract

Lignin is one of the most abundant natural resources that can be exploited for the bioproduction of value-added commodity chemicals. Oil palm empty fruit bunches (OPEFBs), byproducts of palm oil production, are abundant lignocellulosic biomass but largely used for energy and regarded as waste. Pretreatment of OPEFB lignin can yield a mixture of aromatic compounds that can potentially serve as substrates to produce commercially important chemicals. However, separation of the mixture into desired individual substrates is required, which involves expensive steps that undermine the utility of OPEFB lignin. Here, we report successful engineering of microbial hosts that can directly utilize heterogeneous mixtures derived from OPEFB lignin to produce commodity chemicals, adipic acid and levulinic acid. Furthermore, the corresponding bioconversion pathway was placed under a genetic controller to autonomously activate the conversion process as the cells are fed with a depolymerized OPEFB lignin mixture. This study demonstrates a simple, one-pot biosynthesis approach that directly utilizes derivatives of agricultural waste to produce commodity chemicals.

## Introduction

Lignocellulosic biomass is the most abundant renewable resource (Vardon et al., 2015; Deng et al., 2016). In particular, oil palm empty fruit bunches (OPEFBs), byproducts of palm oil production, are abundant lignocellulosic biomass largely burned for energy and regarded as waste. It is estimated that 1.1 tons of OPEFBs are generated per ton of oil palm produced, which amounts to 57 million tons annually (Murphy, 2014; Coral Medina et al., 2015). Given their availability in large quantities, OPEFBs are an attractive renewable lignocellulosic source that can serve as feedstock in biorefineries to produce value-added products. OPEFB can be converted to fermentable sugars (Li et al., 2014) and lignin extract (Mohamad Ibrahim et al., 2008) through simple, cost-effective pretreatments using chemicals and heat.

**FIGURE 1:**
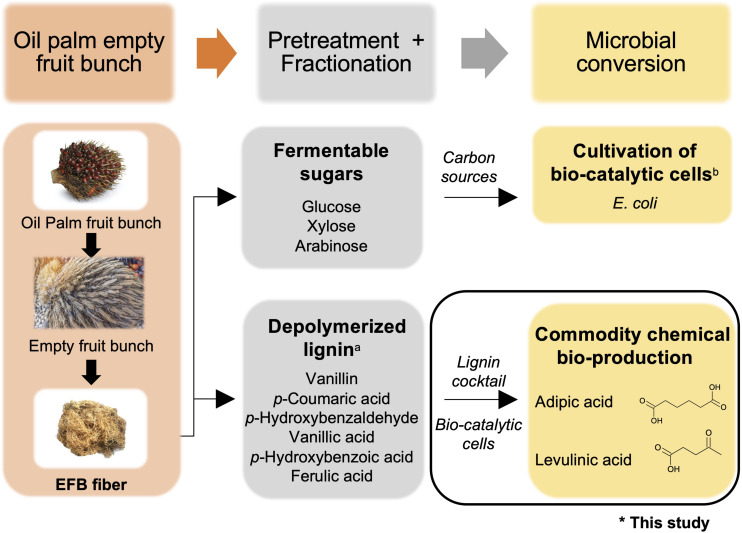
Overview of commodity chemical production using oil palm empty fruit bunches (OPEFBs). The aim of this study is indicated by the black box. ^*a*^Reported in Mohamad Ibrahim et al. (2008); ^*b*^reported in Li et al. (2014). Images are from Shutterstock under paid subscription.

The use of OPEFB-derived fermentable sugars to support cell growth was explored in 2014 by Li et al. (2014) who combined the use of dilute acid and whole fungal cell culture-catalyzed hydrolysis to extract fermentable sugars from OPEFBs. Hemicellulose was first stripped off from OPEFBs using acid hydrolysis, and the remaining cellulose-lignin complex was converted to glucose by the cellulases in the whole fungal cell culture. The OPEFB-derived sugars were subsequently used as a carbon source to cultivate *Escherichia coli* (*E. coli*) as a proof of concept. The use of OPEFB-derived lignin was explored in 2008 by Mohamad Ibrahim et al., where lignin was extracted from OPEFBs using 20% sulfuric acid, followed by nitrobenzene oxidation to break down the lignin. This extraction process released a plethora of depolymerized lignin compounds, notably vanillin, *p*-coumaric acid, *p*-hydroxybenzaldehyde, vanillic acid, *p*-hydroxybenzoic acid and ferulic acid (Xu et al., 2012). Vanillin and *p*-coumaric acid were the predominant degradation products, with concentrations of ∼1800 ppm (1.8 g/L) and ∼1000 ppm (1.0 g/L), respectively (Mohamad Ibrahim et al., 2008). These compounds are useful substrates for the production of commercially important organic acids. Taken together, the reported studies suggest that OPEFB derivatives can be potentially exploited for biorefinery processes, and microbial cells can be engineered to convert aromatic compounds into commodity chemicals while utilizing fermentable sugars for cell growth (Figure 1). However, the effective utilization of depolymerized lignin is hampered by the need for a fractionation process for further valorization as value-added chemicals. There are tremendous efforts currently devoted to the development of efficient fractionation processes, although these costly processes can potentially undermine the economic feasibility and utility of OPEFB lignin.

Herein, a proof-of-concept microbial-based bioprocess was devised that directly utilized unfractionated depolymerized OPEFB lignin as a substrate for commodity chemical production (Figure 1). Toward this goal, we engineered *E. coli* to demonstrate feasibility by having synthetic metabolic pathways to serve as a multi-substrate biocatalyst that can directly convert all 6 compounds derived from depolymerized OPEFB lignin into a key precursor intermediate, β-ketoadipic acid, via the β-ketoadipic acid pathway (Wells and Ragauskas, 2012), and subsequently convert this intermediate into various commercially important derivatives such as adipic acid and levulinic acid. Adipic acid is a high-demand commodity chemical that is used as a lubricant and as a precursor for nylon 6,6, polyester polyols and plasticizers (Vardon et al., 2015). It has a market volume of 2.6 million tons per year (Polen et al., 2013) and was valued at USD 5.56 billion in 2016 (Grand View Research, 2018). On the other hand, levulinic acid had a market value of USD 164 million in 2020 (MarketWatch, 2020) and is a versatile chemical having roles in industrial products such as resins, plasticizers, textiles, animal feed, coatings, and antifreeze (Ghorpade and Hanna, 1997).

To further improve the bioconversion and simplify the bioprocess, *E. coli* cells were engineered to have a regulatory element that functions as a dynamic sensor-based genetic controller. In engineered cells, the expression of enzymatic pathway genes is commonly controlled by an induction system, where an artificial inducer is used to activate enzyme expression. The use of such inducers, although effective, is less favorable due to their high cost and toxicity and the corresponding increased bioprocess complexity. To this end, a two-layer genetic controller (Lo et al., 2016) was employed that regulated the enzyme expression and hence bioconversion based on the availability of nutrient and OPEFB lignin derivatives. This enabled the engineered *E. coli* to autonomously activate the bioconversion process when the substrates are available without the need for additional inducer.

Taking these results together, we demonstrated the direct utilization of OPEFB lignin derivatives using engineered *E. coli* to produce a precursor intermediate, β-ketoadipic acid. Biosynthesis of commodity chemicals using this intermediate was also demonstrated, where up to 9.5 mg/L adipic acid and 455.57 mg/L levulinic acid were produced from the reconstituted OPEFB lignin cocktail under fermenter-controlled conditions.

## Materials and Methods

### Plasmid Assembly

The plasmid backbones used in this study were the BglBrick vectors (Lee et al., 2011) pBbE8k and pBbE8a from the Joint BioEnergy Institute, United States. Cloning and modification of DNA parts such as promoters, genes, and terminators required the use of the splicing overlap extension (SOE) technique (Heckman and Pease, 2007). Biological parts were either PCR-cloned from genomic templates of *P. putida* KT2440 and *E. coli* K-12 MG1655 or SOE-assembled using gene fragments (gBlocks) from Integrated DNA Technologies, United States. They were converted into the BglBrick standard, which comprised of universal linkers such as EcoRl, *Bgl*II, *Bam*HI and Xhol restriction sites for assembly. The standard BglBrick assembly method, described by Anderson et al. (2010) (Anderson et al., 2010), was used to assemble the genetic constructs listed in Supplementary Figure 1. Recombinant BglBrick plasmids were chemically transformed into *E. coli* K-12 TOP10 (Invitrogen, United States). Transformed *E. coli* strains were first cultivated in Luria-Bertani (LB) broth at 37°C and 225 rpm and screened via colony PCR. Gene deletion was introduced using a previously described method (Datsenko and Wanner, 2000). List of bacterial strains and plasmids used in this study are listed in Supplementary Table 1.

### Preparation of Cell Extracts for *in vitro* Enzymatic Assays for Adipic Acid Pathway Validation

*Escherichia coli* BL21(DE3) was transformed with each plasmid bearing one of the PcaI-, PcaJ-, PaaH-, Ech-, egTer-, tdTer-, Ptb-, or Buk1-encoding genes in either pBbE8k or pACYCDuet-1 (Novagen, Germany). Each gene was derived from *P. putida* KT2440 (PcaI and PcaJ), *Ralstonia eutropha* (PaaH1), *R. eutropha* H16 (Ech), *Euglena gracilis* (egTer), *Treponema denticola* (TdTer) or *Clostridium acetobutylicum* (Ptb and Buk1). The seed cultures for the transformants were prepared by overnight cultivation in LB medium supplemented with the appropriate antibiotic (30 μg/L kanamycin or 50 μg/L ampicillin) at 37°C and 225 rpm. The seed cultures were diluted 1:100 (v/v) into Terrific Broth medium supplemented with appropriate antibiotics (30 μg/L kanamycin or 50 μg/L ampicillin) and cultivated at 37°C and 225 rpm. The diluted *E. coli* cultures were induced with 0.1 mM IPTG at OD_600_ 0.5 - 1.0 and cultivated at 16°C and 225 rpm for 24 h. The cultures were harvested and resuspended with 0.5 mL of lysis buffer (20 mM Tris–HCl, 200 mM NaCl, 1 mM DTT, and 10% (v/v) glycerol, pH 7.5, final concentrations) and incubated at 25°C and 150 rpm for 1 h with 1.5 mg/ml lysozyme. After the addition of 0.1% Triton X-100 and 1x protease inhibitor (Promega), the soluble fraction of crude cell extract was prepared by using FastPrep-24^TM^ 5G (MP Biomedicals) and acid-washed beads (≤ 106 μm) (Sigma-Aldrich) at 6.5 m/s and 45 s, followed by centrifugation at 4°C and 13,000 rpm for 10 min. Total proteins in the soluble extracts were manually quantified by using Bradford reagent (Sigma-Aldrich). The overexpression of each gene was verified by SDS–PAGE.

### *In vitro* Enzyme Assay for β-Ketoadipic Acid Succinyl-CoA Transferases

The activities of β-ketoadipic acid succinyl-CoA transferase (subunit, PcaI and subunit, PcaJ) were determined as described previously (MacLean et al., 2006), with slight modifications. Briefly, the reaction was started with the addition of a reaction mixture (200 mM Tris–HCl, 0.4 mM succinyl-CoA, 40 mM MgSO_4_, and 1 g/L β-ketoadipic acid, pH 8.0, final concentrations) to aliquots of cell extracts to a final volume of 0.1 mL. The formation of β-ketoadipyl-CoA:Mg^2+^ was monitored at 305 nm and 30°C for 4 min by using a Biotek Synergy H1m microplate reader.

### *In vitro* Adipate Production

*In vitro* adipate production was performed as described previously (Yu et al., 2014), with the following modifications. Each cell extract (equivalent to 0.05 mg of total protein) for PcaI, PcaJ, PaaH1, Ech, Ter (either egTer or tdTer), Ptb, and Buk1 was added into a reaction mixture (50 mM potassium phosphate buffer, 0.4 mM succinyl-CoA, 4 mM NADH, 2 mM ADP, and 0.5 g/L β-ketoadipic acid, pH 7.0, final concentrations) to a final volume of 0.2 mL and incubated for 24 h at room temperature. Subsequently, each sample was mixed with 0.2 mL of 1 M HCl and internal standard (1,14-tetradecanedioic acid) to a volume of 0.5 mL and vortexed thoroughly for 30 s. After the addition of 0.5 mL of ethyl acetate, the sample was vortexed thoroughly for 1 min, followed by centrifugation at 13,000 rpm for 1 min. Then, 0.35 mL of the ethyl acetate fraction was aliquoted and evaporated by using a rotary evaporator, followed by resuspension in 0.04 mL of ethyl acetate. The resuspended sample was mixed with N,O-bis(trimethylsilyl)trifluoroacetamide (BSTFA) at a 1:1 (v/v) ratio and derivatized at room temperature for 24 h. The formation of adipic acid was analyzed using GC-MS.

### Shake-Flask Adipic Acid Production for Engineered Host Screening

Overnight seed cultures were diluted 1:100 (v/v) into 50 mL of M9 medium supplemented with 0.2% (w/v) glucose, 0.2% (w/v) casamino acids and the appropriate antibiotics (100 μg/L carbenicillin, 50 μg/L kanamycin, and 25 μg/L chloramphenicol) in 250 mL baffled flasks and cultured at 30°C and 225 rpm. The engineered *E. coli* cultures were induced with 0.2% (w/v) L-arabinose at OD_600_ 1.2 - 1.5, which was followed by the addition of the *p*-coumaric acid substrate to a final concentration of 0.1% (w/v). Sampling was performed at 18h, 36h and 42h. The formation of adipic acid was analyzed by using GC-MS.

### Bioconversion by Engineered Cells Bearing Different Controllers

Engineered *E. coli* MG1655 cells were first grown in M9 medium (supplemented with 0.2% (w/v) glucose and 0.2% (w/v) casamino acids) to the exponential phase at OD_600_ 1.0. The inoculums were added to shake flasks (37°C, 225 rpm) to a final concentration of OD_600_ 0.01, with each flask containing 50 mL of M9 medium supplemented with 0.2% (w/v) glucose as the carbon source, 0.2% (w/v) casamino acids and the relevant lignin derivatives as substrates. *p*-Coumaric acid (Sigma-Aldrich, United States) was first dissolved in dimethyl sulfoxide (DMSO) to a stock concentration of 10% (w/v) before being added to the M9 medium to a final concentration of 0.1% (w/v). The L-arabinose system and HA-controlled system were induced with 0.2% (w/v) L-arabinose or 0.1% (w/v) *p*-coumarate, respectively, after reaching OD_600_ 1.0. One milliliter of the biotransformation culture was extracted at each time point (18h, 36h and 42h) for GC-MS measurements.

### Reconstitution of OPEFB Depolymerized Lignin Cocktail

Oil palm empty fruit bunches depolymerized lignin cocktail was reconstituted based on the identified concentrations of aromatic compounds reported in Mohamad Ibrahim et al. (Mohamad Ibrahim et al., 2008). In brief, individual compounds of OPEFB were prepared separately and subsequently mixed together to give the final concentrations stated in Table 1. All the individual compounds were purchased from Sigma-Aldrich with a purity of > 97% and prepared in DMSO at a concentration that limits DMSO to 1% (v/v) in the final lignin cocktail solution. All the stock solutions of the compounds were kept at 4°C in aliquots prior to use. Ten milliliter of lignin cocktail was prepared to comprise; 1.8 g of vanillin (Cat. No. 94752), 1 g of *p*-coumaric acid (≥ 98% (HPLC), Cat. No. C9008), 320 mg of *p*-hydroxybenzaldehyde (4-hydroxybenzoic acid; ≥ 99%, Cat. No. 240141), 110 mg of vanillic acid (4-hydroxy-3-methoxybenzoic acid; ≥ 97% (HPLC), Cat. No. 94770), 18 mg of *p*-hydroxybenzoic acid (3,4-dihydroxybenzoic acid; ≥ 98%, Cat. No. 37580) and 13 mg of ferulic acid (*trans*-ferulic acid; 99%, Cat. No.128708) in DMSO. The solution was vortexed to ensure homogenized mixture. The cocktail was diluted 100-fold in the reaction volume to result in the final substrate concentration and referred as ‘1x OPEFB.’

**TABLE 1:** Production of protocatechuic acid (PCA) using EFB lignin derivatives as substrates at 36 h.

Substrates	Substrate used (mg)	PCA (mg/L)	% Conversion
Vanillin	1800	49.6 ± 6.7	2.7
*p*-coumaric acid	1000	273.8 ± 41.7	29.1
*p*-hydroxybenzaldehyde	320	166.2 ± 28.9	41.1
Vanillic acid	110	112.5 ± 1.4	100.0
*p*-hydroxybenzoic acid	18	7.2 ± 6.0	36.0
Ferulic acid	13	7.2 ± 1.7	69.6
EFB (All)	3261	380.2 ± 10.2	11.5

*The concentration of substrates used represents the concentration found in the pretreated, depolymerized EFB lignin.*

### Bioconversion by Engineered Cells Using OPEFBs in Batch Culture

Batch fermentation was carried out in a 5 L working volume BIOSTAT^®^ B-DCU II benchtop bioreactor (Sartorius Stedim) for bioconversion to generate adipic acid or levulinic acid. The temperature was maintained at 30°C, and the pH was controlled at 7.0 by automated addition of acid (1 M H_2_SO_4_) and base solutions (1 M NaOH). Oxygen was constantly supplied at 10 L/min, and the impeller speed was set to 400 rpm to ensure homogenous aeration. Antifoam agent (200 μL) was added to the culture to prevent excessive foaming. A seed culture of relevant engineered *E. coli* MG1655 cells was cultured at 30°C overnight and subsequently transferred into 1 L of fresh culture media containing 3 antibiotics (100 mg/L carbenicillin, 50 mg/L kanamycin, and 25 mg/L chloramphenicol). For fermentation of engineered *E. coli* with an L-arabinose-controlled circuit, substrate (OPEFB lignin cocktail) and inducer (0.2% (w/v) L-arabinose) were added 4 h post-inoculation as the culture reached late log phase. For fermentation of engineered *E. coli* with an HA-controlled circuit, the substrate was fed into the vessel immediately following inoculation. Aliquots of samples were taken at 18 h, 36 h and 42 h for further analysis using GC-MS and absorbance measurements at 600 nm. Batch fermentations were run in duplicate, and the results are reported as the average with standard deviation.

### HPLC Quantification

Ferulic acid, p-coumaric acid, vanillin, vanillic acid, p-hydroxybenzaldehyde, p-hydroxybenzoic acid, and protocatechuic acid quantification were carried out using the protocol adopted by Barghini et al. (Barghini et al., 2007) with modifications. First, 0.4 mL of the extracted batch culture was filter-sterilized with a 0.22 μm filter (Sartorius *Stedim, Germany)* before analysis by an Agilent 1260 HPLC apparatus equipped with an Inertsil ODS3 C18 reverse-phase column (length 250 mm, diameter 4.6 mm and particle size 5 μm) and a diode array detector (DAD). Compounds in the filtered culture were eluted with an isocratic pressure of 150 bars, a mobile phase comprising an aqueous solution of 35% methanol and 1% acetic acid, and a flow rate of 1 mL/min. Detection was performed at UV wavelengths of 300 nm (ferulic acid, *p*-coumaric acid, vanillin, vanillic acid, *p*-hydroxybenzaldehyde) and 254 nm (*p*-hydroxybenzoic acid, protocatechuic acid) with a sample injection volume of 10 μl. The retention times of the samples were compared with those of purified standards (*Sigma-Aldrich, United States*) for identification and quantification.

### Gas Chromatography - Mass Spectrometry (GC-MS) Identification and Quantification

To extract organic acids (β-ketoadipic acid, adipic acid and levulinic acid) for detection, 500 μL of 1 M HCl, 300 μL of ethyl acetate and 100 μL of an internal standard (1,14-tetradecanedioic acid) were added to 1 ml of a cell culture sample. Subsequently, cells were disrupted by bead beating (FastPrep-24^TM^ 5G and acid-washed beads (≤ 106 μm) running at 6.5 m/s and 1 min interval 4 times) and centrifuged for 10 min at 20,000 x g at 4°C to separate the organic phase. The ethyl acetate extracts were incubated with a derivatization agent [BSTFA with 1% trimethylchlorosilane (TCMS)] overnight prior to analysis by gas-liquid chromatography (GC) using an Agilent 7890B GC system equipped with an HP-5MS column (Agilent) coupled to a mass spectrometer (Agilent 5977).

## Results and Discussion

### An Enzymatic Pathway Enabling OPEFB Lignin Utilization

**FIGURE 2:**
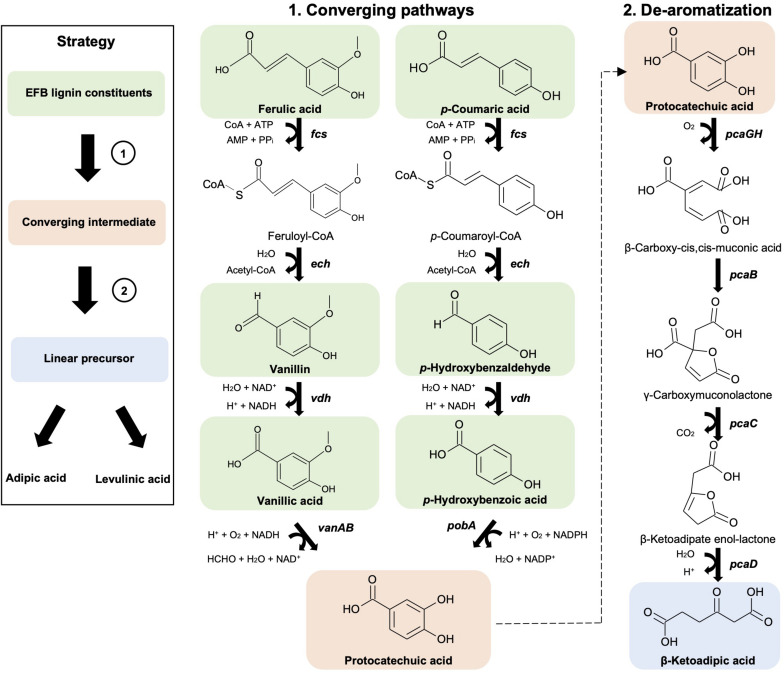
Synthetic metabolic pathways involved in the conversion of OPEFB lignin derivatives to converge to a single intermediate (protocatechuic acid) (1) and a linear precursor (β-ketoadipic acid) (2) that are needed for adipic acid and levulinic acid production. Pathway genes (in bold) and enzymes are ***fcs*** (feruloyl-CoA synthetase), ***ech*** (enoyl-CoA hydratase), ***vdh*** (vanillin dehydrogenase), ***vanAB*** (vanillate O-demethylase), ***pobA*** (*p*-hydroxybenzoate hydroxylase), ***pcaGH*** (protocatechuate 3,4-dioxygenase), ***pcaB*** (3-carboxy-cis,cis-muconate cycloisomerase), ***pcaC*** (4-carboxymuconolactone decarboxylase) and ***pcaD*** (β-ketoadipate enol-lactone hydrolase). Chemical structures were drawn using ACD/ChemSketch.

As a first step to convert the depolymerized OPEFB lignin to value-added chemicals, a 9 enzyme pathway (Figure 2, Supplementary Figure 1d) derived from *Pseudomonas putida* KT2440 was designed and assembled in *Escherichia coli* K-12 MG1655, a workhorse of industrial biotechnology. The ability of the *E. coli* K-12 MG1655 strain containing this metabolic pathway to utilize all OPEFB lignin derivatives, convert them into the single compound protocatechuic acid and subsequently convert that to a linear precursor, β-ketoadipic acid, was examined.

First, a converging pathway was constructed that comprises feruloyl-CoA synthetase (Fcs), enoyl-CoA hydratase (Ech), vanillin dehydrogenase (Vdh), vanillate O-demethylase (VanAB), and *p*-hydroxybenzoate hydroxylase (PobA) (Supplementary Figure 1c). To demonstrate the feasibility of the constructed pathway functioning in the microbial host, the bioconversion of individual lignin substrates for protocatechuic acid production was validated first (Figure 2 and Table 1), followed by bioconversion of OPEFB lignin derivatives. The results show that the converging pathway is able to utilize all of the OPEFB lignin derivatives, i.e., vanillic acid, ferulic acid, *p*-hydroxybenzaldehyde, *p*-coumaric acid, *p*-hydroxybenzoic acid and vanillin (in descending order of conversion efficiency), and convert them into the single intermediate molecule protocatechuic acid. The most efficient conversion was observed with vanillic acid and ferulic acid, producing ∼100% and ∼70% of the theoretical yields, respectively (Table 1).

When the OPEFB lignin derivatives (formulated in the ratio that is naturally found after the pretreatment) were tested, up to 400 mg/L protocatechuic acid was detected, reaching 11.5% of the theoretical yield. The less-than-expected yield is mostly due to the inefficient utilization of vanillin, where only 2.7% molar conversion to protocatechuic acid was observed despite its high starting concentration (1.8 g/L). As a high concentration of vanillin has been reported to inhibit bacterial growth (Zaldivar et al., 1999), one possible approach to improve vanillin utilization would be to oxidize the depolymerized OPEFB lignin mixture, especially vanillin (Fargues et al., 1996), to the less toxic compound vanillic acid prior to its feeding to the engineered cells. As vanillic acid has shown complete conversion, this approach may improve the yield of bioproduction and reduce toxicity to the host cells. However, these approaches have not been fully explored in this study, as the aim of this work is to first demonstrate the feasibility of direct conversion of the OPEFB lignin cocktail.

After the successful production of protocatechuic acid from the OPEFB lignin derivatives, a de-aromatization pathway involving protocatechuate 3,4-dioxygenase (PcaGH), 3-carboxy-cis,cis-muconate cycloisomerase (PcaB), 4-carboxymuconolactone decarboxylase (PcaC) and β-ketoadipate enol-lactone hydrolase (PcaD) (Figure 2) was shown to function together with the organic acid production pathway in the subsequent experiment.

### *De novo* Organic Acid Production Pathways From β-Ketoadipic Acid in *E. coli*

**FIGURE 3:**
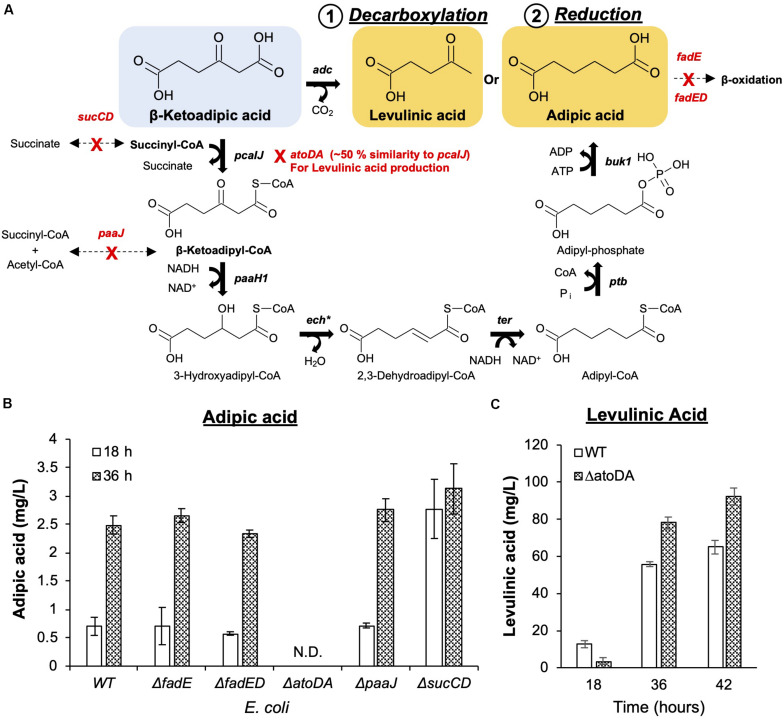
Commodity chemical production from β-ketoadipic acid. **(A)** Synthetic metabolic pathways to convert the linear precursor (β-ketoadipic acid) to levulinic acid (1. Decarboxylation) and adipic acid (2. Reduction). Native enzymes that can potentially compete with the pathways have been identified and deleted from the working host strain *E. coli* MG1655. Pathway genes (in bold) and enzymes are ***pcaIJ*** (3-ketoacetate CoA transferase), ***paaH1*** (3-ketoadipoyl-CoA reductase), ***ech****^∗^* (enoyl-CoA hydratase), ***ter*** (2,3-dehydroadipoyl-CpA reductase), ***ptb*** (phosphate butyryltransferase), ***buk1*** (butyrate kinase 1) and ***adc*** (acetoacetate decarboxylase). ***ech***^∗^ is a different enoyl-CoA hydratase from the one used in the protocatechuic acid pathway. **(B)** Deletion strains were evaluated for adipic acid production. The deleted genes are ***fadE*** (acyl-CoA dehydrogenase), ***fadD*** (long-chain fatty acid CoA ligase), ***paaJ*** (β-ketoadipyl-CoA thiolase), and ***sucCD*** (succinyl-CoA synthetase). **(C)** The *E. coli* Δ*atoDA* strain was selected as it ensures metabolic flux toward levulinic acid production. Images are generated using MS Office softwares and ACD/ChemSketch.

A direct biosynthesis of adipic acid from carbon sources in *E. coli* has been reported (Yu et al., 2014; Cheong et al., 2016; Zhao et al., 2018) where artificial adipic acid synthesis pathways were constructed to convert glucose or glycerol to adipic acid. In a recent study, Niu et al. (2020) successfully demonstrated adipic acid production from β-ketoadipic acid in *Pseudomonas putida* KT2440. Adapted from these findings, an adipic production pathway was constructed and validated (Supplementary Figure 2A) in *E. coli*. The constructed pathway utilizes β-ketoadipic acid by (1) esterifying it with CoA by β-ketoadipic acid succinyl-CoA transferase (PcaIJ); (2) subsequently reducing the 3-oxo group by 3-hydroxyacyl-CoA dehydrogenase (PaaH1), enoyl-CoA hydratase (Ech), and trans-enoyl-CoA reductase (Ter); and (3) removing CoA by phosphate butyryltransferase (Ptb) and butyrate kinase 1 (Buk1) to form adipic acid. To test this entire pathway, the 6 enzymes were first individually expressed in *E. coli* BL21(DE3) (Supplementary Figure 2A) and subsequently characterized for their activity. *In vitro* enzymatic activities for β-ketoadipic acid degradation and 3-oxo reduction to adipic acid (Supplementary Figures 2B,C) were measured. We observed that 3-oxo reduction required screening for a suitable reducing enzyme, Ter, that is responsible for converting 2,3-dehydroadipyl-CoA to adipyl-CoA. Adipic acid (1.18 mg/L) was detected only when Ter from *Treponema denticola* (TdTer) was used and not when the Ter from *Euglena gracilis* (EgTer) was used. Through this systematic *in vitro* enzymatic characterization, we verified the novel enzymatic pathway from β-ketoadipic acid to adipic acid (Figure 3A).

Unlike the adipic acid pathway, levulinic acid production involves a single decarboxylation step from β-ketoadipic acid. This reaction was catalyzed by acetoacetate decarboxylase (*Adc*) from *Clostridium acetobutylicum* (Cheong et al., 2016). The level of levulinic acid exceeded 60 mg/L within 36 h of bioconversion under shake-flask conditions (Figure 3C).

### Host Engineering for Optimizing Value-Added Chemical Production

To facilitate the conversion of β-ketoadipic acid, repurposing of the existing native metabolic pathway in *E. coli* was required to channel reduction and decarboxylation pathways (Figure 3A). This involved using the *E. coli* K-12 MG1655 reference genomic model in the EcoCyc database (Keseler et al., 2016) to search for potential native genes that may be able to divert intermediates or cofactors to other products. We postulated that native genes of *E. coli* may compete and negatively impact the designed pathways, namely, *fadE*, *fadED*, *paaJ*, and *sucCD* for adipic acid production and *atoDA* for levulinic acid production. We assessed the impact of each gene deletion based on bioconversion of *p*-coumaric acid and used the amount produced as an indicator for pathway repurposing efficiency (Figures 3B,C).

For adipic acid production, acyl-CoA dehydrogenase (*fadE)* and long-chain fatty acid CoA ligase (*fadD*) or both (*fadED)* genes were targeted, as these are involved in fatty acid metabolism (Lennen et al., 2010; Sathesh-Prabu and Lee, 2015). This metabolism can potentially utilize six-carbon dicarboxylic acid (adipic acid) for β-oxidation (Smit et al., 2005). However, the deletion of these genes did not significantly improve adipic acid production. As succinyl-CoA is an important cofactor in the formation of β-ketoadipyl-CoA, *sucCD* (*sucC* and *sucD*, which encode subunits of succinyl-CoA synthetase) were deleted to minimize the competing conversion of succinyl-CoA towards succinate (Birney et al., 1996; Zhao et al., 2018). β-Ketoadipyl-CoA thiolase (*paaJ*) is also targeted for deletion due to its role in the reversible catalysis of β-ketoadipyl-CoA to succinyl-CoA and acetyl-CoA (Yu et al., 2014; Babu et al., 2015). Among the list of genes that can potentially shunt intermediates from the introduced reduction reactions, *sucCD* deletion resulted in the greatest improvement in adipic acid production. Although the production titer for all mutants and wild-type strain was similar at 36h, *sucCD* mutant was able to convert the substrates at an approximately 3-fold higher efficiency than other mutants, as shown by the higher production observed at an early time point (18 h) (Figure 3B).

For levulinic acid production, as acetoacetate decarboxylase (Adc) (Cheong et al., 2016) was used to convert β-ketoadipic acid, the *atoDA* genes from the acetoacetic acid degradation pathway in *E. coli* were targeted for deletion. The *atoDA* genes were targeted because they share > 50% amino acid sequence similarity with the *pcaIJ-*encoded enzyme based on sequence alignment (Supplementary Figure 3). Based on the similarity, the deletion was anticipated to promote the intended decarboxylation. Indeed, the *atoDA*-deleted strain was able to promote an approximately 40% increase in levulinic acid compared to its level in wild-type *E. coli* (Figure 3C).

Overall, the outcome of the host engineering approaches indicates that the *sucCD*-deleted host is suitable for adipic acid production and that the *atoDA*-deleted host is suitable for levulinic acid production. These two strains were used in subsequent experiments for downstream optimization.

### Genetic Controller Enabling Autonomous OPEFB Lignin-Derived Hydroxycinnamic Acid-Dependent Regulation

**FIGURE 4:**
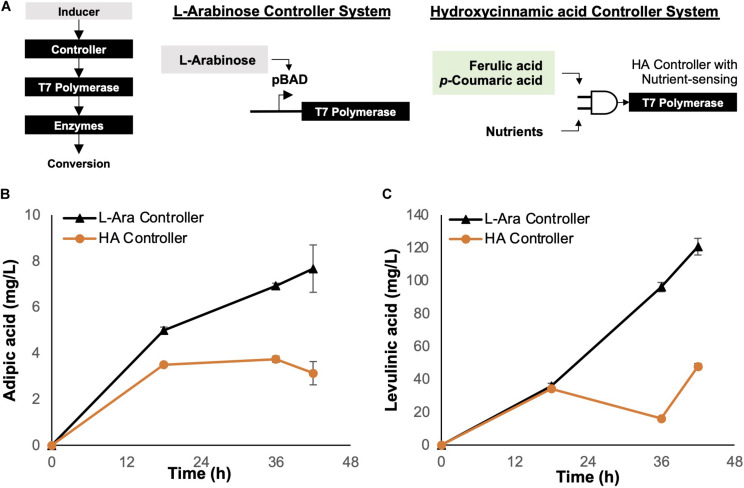
Controllers for enzyme regulation. **(A)** Genetic circuit controller systems. The L-arabinose (arabinose-inducible) controller system was compared with the hydroxycinnamic acid (lignin substrate-inducible) controller system. The genetic controller turns on expression of T7 polymerase, which in turn controls the expression of enzymes required for bioconversion of depolymerized lignin. **(B)** Bioconversion of *p*-coumarate to adipic acid using *E. coli* Δ*sucCD* with a controller system regulating enzyme expression. **(C)** Bioconversion of *p*-coumarate to levulinic acid using *E. coli* Δ*atoDA* with a controller system regulating enzyme expression. Images are generated using MS Office softwares and ACD/ChemSketch.

During pathway validation and host engineering experiments, an induction system based on the L-arabinose (Guzman et al., 1995) inducer was used to regulate the expression of pathway enzymes in a dose-dependent manner. The role of the genetic controller is to modulate downstream gene transcription through the expression of the bacteriophage-based T7 polymerase (Figure 4A). Since the organic acid production pathways are long (15 enzymes for adipic acid production and 10 enzymes for levulinic acid), to ensure good transcription of all the genes, the genes were placed under the non-native, strong T7 promoter, which is recognized by the T7 polymerase to initiate downstream transcription. However, uncontrolled expression of the T7 polymerase can lead to overexpression of target proteins, which may burden the host cell (Kesik-Brodacka et al., 2012); hence, expression must be regulated by a genetic controller that restricts T7 polymerase transcription via external inputs such as chemical inducers.

Although the L-arabinose controller is an effective genetic device, it requires additional external resources, i.e., L-arabinose acts as an inducer, thus increasing the deployment cost of the biocatalytic cells. To improve the economics of OPEFB lignin utilization, we considered employing the hydroxycinnamic acid (HA) controller system reported by Lo et al. (2016). The HA controller system is inducible by HAs such as ferulic acid and *p*-coumaric acid, which are present in the depolymerized OPEFB lignin.

For comparison, we tested both L-arabinose and HA controllers for adipic and levulinic acid production in optimized host strains (Δ*sucCD* and Δ*atoDA*, respectively) under shake-flask conditions at 30°C and using *p*-coumaric acid (final concentration, 1 g/L) as the substrate (Figure 4). The fermentation temperature was set at 30°C instead of the commonly used 37°C based on two reasons: (1) less energy is required to maintain a lower temperature while not impacting the growth of the engineered *E. coli*, and (2) the unstable compound β-ketoadipic acid can have a longer half-life for enzymatic conversion at the lower temperature. The L-arabinose-induced controller performed better than the HA controller in terms of the product yield: a 2-fold higher titer was observed for both adipic and levulinic acid production (Figures 4B,C). We hypothesized that the concentration of the inducer L-arabinose (0.2% w/v) used in the shake-flask experiment resulted in rapid overexpression of enzymes in a given time frame, resulting in higher bioconversion than the HA controller.

### Optimized Host With Hydroxycinnamic Acid Controller for Efficient OPEFB Lignin Utilization and Bioconversion

**FIGURE 5:**
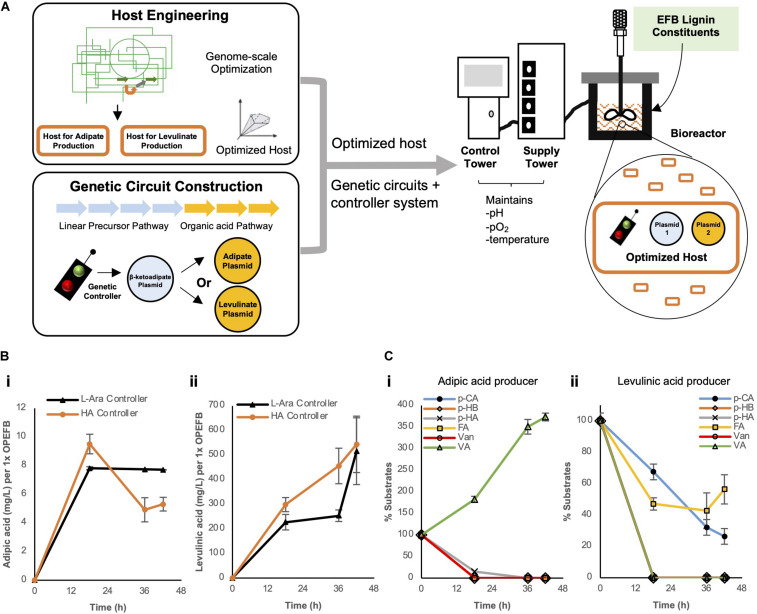
**(A)** Overall strategies used to improve biosynthesis of adipic or levulinic acid by engineered microbes using depolymerized EFB lignin derivatives. In bioreactors, **(B)** the biosynthesis of adipic acid and levulinic acid and **(C)** utilization of depolymerized OPEFB lignin derivatives were quantified. The 6 aromatic compounds are converted to protocatechuic acid (PCA) as outlined in the converging pathways in Figure 2. pCA: *p-*coumaric acid, pHB: *p*-hydroxybenzaldehyde, pHA: *p-*hydroxybenzoic acid, FA, Ferulic acid; Van, Vanillin; VA, Vanillic acid. Images are generated using MS Office softwares and ACD/ChemSketch.

In an attempt to further boost the yield, we decided to overcome the inherent problems faced by shake-flask experiments, such as limited oxygen levels and uncontrolled pH, which can affect the productivity of microbial hosts. This was achieved by using controlled bioreactor which can regulate these parameters during fermentation process. Bioreactors with oxygen (pO_2_) and pH sensors and their relevant pumps to maintain these parameters at target values were used for OPEFB lignin conversion (Figure 5A). The concerns for oxygen availability and the need for pH regulation are due to (i) the need for oxygen for cellular metabolic growth and de-aromatization of protocatechuic acid and (ii) CO_2_ generation during adipic or levulinic acid production that leads to a decreasing pH in the cell culture over time. To further improve the bioconversion of the OPEFB lignin cocktail by the engineered cells, we optimized the feed dosage to the bioreactors (Supplementary Figure 4 – 0.5x OPEFB lignin for levulinic acid conversion and 0.375x OPEFB lignin for adipic acid conversion).

Under controlled conditions, the respective optimized host strains (Δ*sucCD* and Δ*atoDA*) bearing the HA controller performed similar to, if not slightly better, than the strains bearing the L-arabinose controller: ∼1.8-fold higher titer was observed for levulinic acid production (455.7 mg/L vs. 253.5 mg/L per 1x OPEFB lignin at 36 h) and ∼23% higher for adipic acid production (9.5 mg/L vs 7.8 mg/L per 1x OPEFB lignin at 18 h) in the HA controller strains (Figure 5B). Given the modest production of adipic acid in our engineered cells, to identify a potential rate-limiting step(s) in our synthetic metabolic pathways (depicted in Figure 2), we quantified the key substrates of the synthetic metabolic pathways in levulinic and adipic acid producing cells over time (Figure 5C). This measurement shows a significant accumulation of vanillic acid in the adipic acid producing cells, suggesting that the enzymatic conversion of vanillic acid be a major rate-limiting step. In the levulinic acid producing cells, *p*-coumaric acid and ferulic acid were not fully utilized (Figure 5C), which represent rate-limiting steps in the synthetic metabolic pathways. This result suggests that the production of levulinic acid and adipic acid in our engineered cells can be further increased if the aforementioned rate-limiting enzymatic reactions are improved.

Overall, this is the first report of autonomous cell-based production of adipic and levulinic acid from an OPEFB lignin cocktail without the need for upstream separation into individual derivatives before conversion and costly chemical inducers. In this study, we evaluated our conceptual strategy by producing adipic acid and levulinic acid from OPEFB lignin, primarily because both are industrially relevant chemicals that can be derived from the versatile dearomatized precursor β-ketoadipic acid. The implications of the strategy of converging various lignin substrates into β-ketoadipic acid and subsequently deriving the desired product can be further extensively explored. For instance, any kind of downstream organic acid synthesis pathway could be coupled with our HA-sensing genetic controller and β-ketoadipic acid pathway to produce other economically useful chemicals, such as methyl ethyl ketone (MEK) and 2-butanol. MEK is used as a solvent for paints, coatings, adhesives and inks, while 2-butanol is used as a biodiesel additive.

## Conclusion

In this study, we demonstrate direct utilization of unfractionated depolymerized OPEFB lignin to produce commodity chemicals using an engineered *E. coli* strain. *E. coli* was engineered to have 3 genetic modules for the following functions: 1. genetic control for autonomous activation, 2. conversion of depolymerized lignin derivatives into β-ketoadipic acid by pathway enzymes, and 3. conversion of β-ketoadipic acid to commodity chemicals by pathway enzymes. As a proof of concept, we demonstrated the production of adipic acid and levulinic acid using engineered *E. coli*, where up to 9.5 mg/L adipic acid and 455.57 mg/L levulinic acid were produced from reconstituted OPEFB lignin derivatives in fermenter-controlled conditions. Although our experiment used reconstituted OPEFB lignin, showing moderate titer and yield, the results of our research demonstrate a simple, one-pot biosynthesis approach that potentially be used to directly utilize derivatives of agricultural waste to produce commodity chemicals. This finding provides a strong foundation for future pathway and process optimizations that are required to achieve practical application of microbial cell factories for OPEFB lignin valorization.

## Data Availability Statement

The raw data supporting the conclusions of this article will be made available by the authors, without undue reservation.

## Author Contributions

T-ML, IH, and H-SC designed and performed the experiments and analyzed experimental data. RF and SC performed the experiments. WC and MC oversaw the project and provided guidance. T-ML, IH, and MC wrote, reviewed, and edited the manuscript. All authors have read and agreed to the published version of the manuscript.

## Conflict of Interest

The authors declare that the research was conducted in the absence of any commercial or financial relationships that could be construed as a potential conflict of interest.
