# Supplementary material for: Biosynthesis of Commodity Chemicals From Oil Palm Empty Fruit Bunch Lignin
Source: Front Microbiol. 2021 Apr 9;12:663642. doi: 10.3389/fmicb.2021.663642 (PMC8064122; doi:10.3389/fmicb.2021.663642)
Supplement: Supplementary file 2 [file Table_2.DOCX]

Supplementary Material

## Supplementary Figures


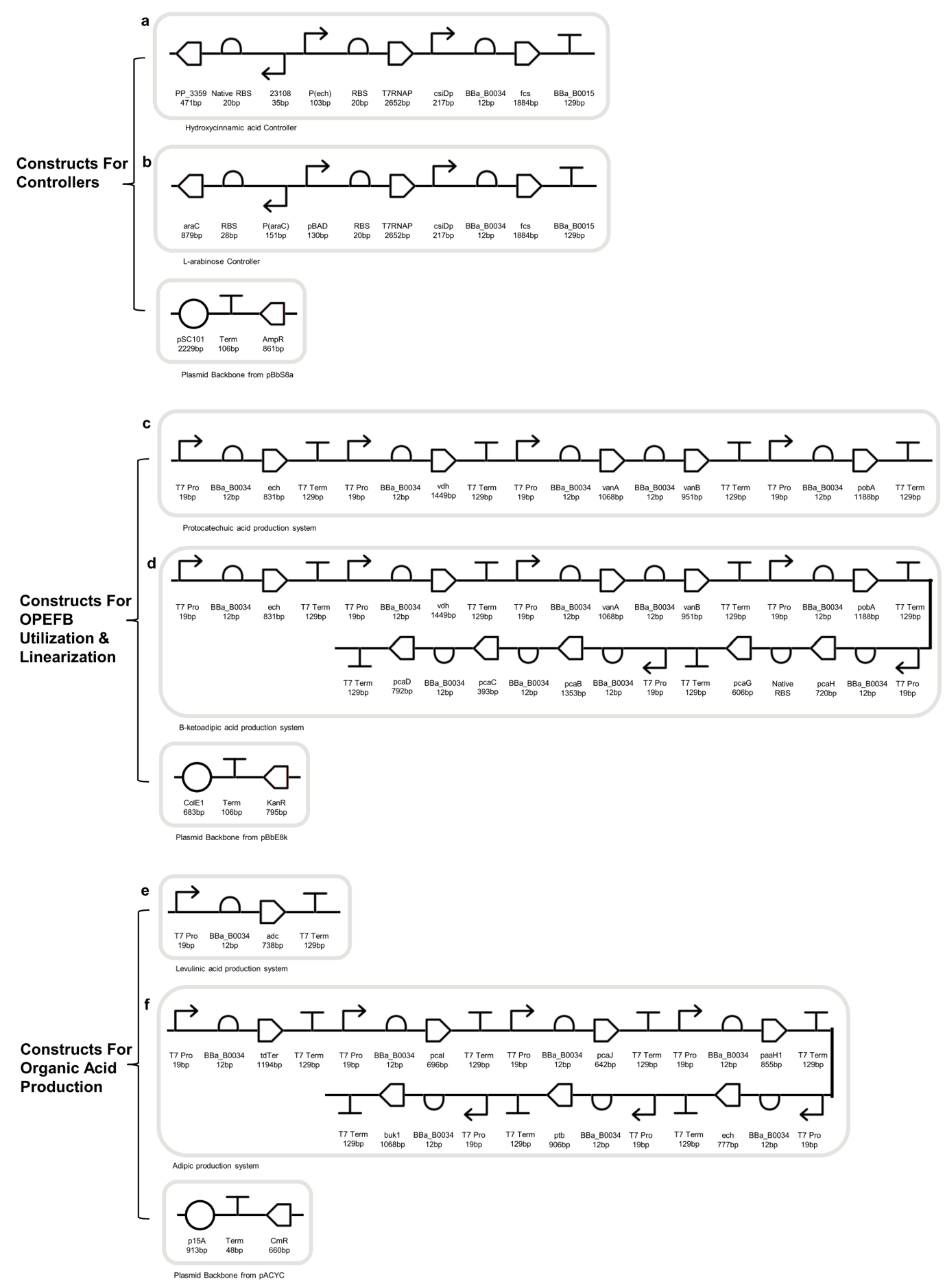


**Supplementary Figure 1. Genetic constructs used in this study.**


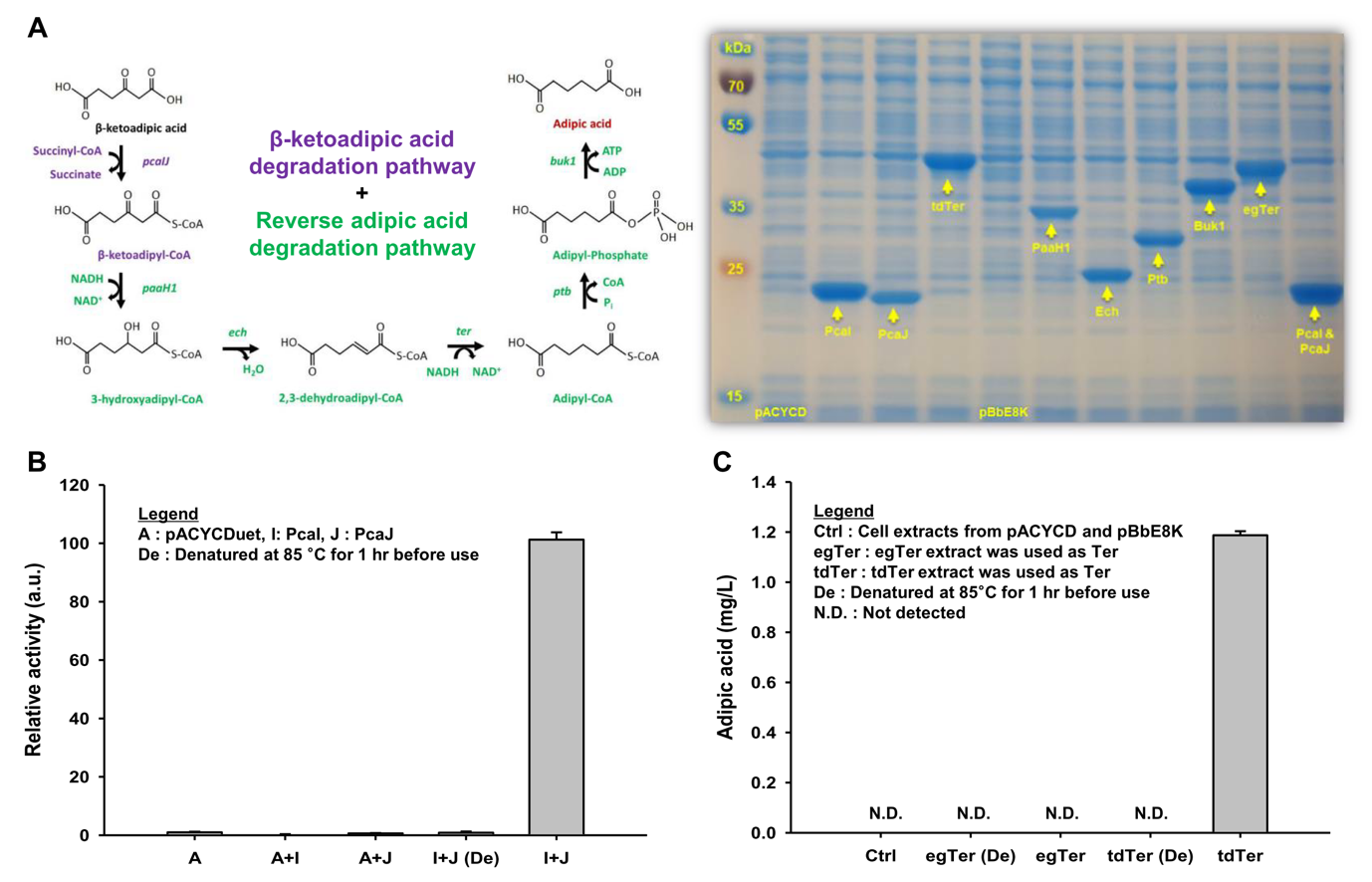


**Supplementary Figure 2. Construction and validation of a novel metabolic pathway to produce adipic acid using β-ketoadipic acid in *E. coli*.** (A) Biosynthesis pathway for adipic acid in *E.coli*, and expression of the pathway enzymes. (B). *In vitro* enzyme assay for β-ketoadipic acid succinyl-CoA transferases (PcaI and PcaJ), where formation of β-ketoadipyl-CoA:Mg^2+^ was measured at 305 nm and normalized to control and shown in relative activity (a.u.) (C). Activity of trans-enoyl-CoA reductases (Ter) was characterized by measuring the final production level of adipic acid.

**Supplementary Figure 3. Alignment of PcaIJ and AtoDA amino acid sequences.**

**Supplementary Figure 4. Optimizing the OPEFB lignin feed through balancing cell growth (indicated by OD_600_) and production titer of adipic acid or levulinic acid at the given time points.**

**Supplementary Figure 5**. **Production titer of adipic acid using single substrate (p-coumarate) or OPEFB lignin cocktail (reconstituted).**
